# Decoding the relationship between cow’s milk proteins and development of type 1 diabetes mellitus

**DOI:** 10.20945/2359-4292-2023-0248

**Published:** 2024-08-13

**Authors:** Luís Jesuino de Oliveira Andrade, Gabriela Correia Matos de Oliveira, Luísa Correia Matos de Oliveira, Alcina Maria Vinhaes Bittencourt, Yvana Baumgarth, Luís Matos de Oliveira

**Affiliations:** 1 Departamento de Saúde Universidade Estadual de Santa Cruz Ilhéus BA Brasil Departamento de Saúde, Universidade Estadual de Santa Cruz, Ilhéus, BA, Brasil; 2 Programa Saúde da Família Salvador BA Brasil Programa Saúde da Família, Salvador, BA, Brasil; 3 Polytech Nancy France Ecole Supériuere des Sciences et Technologies de I’Ingénie de Nancy – Polytech Nancy, France; 4 Faculdade de Medicina Universidade Federal da Bahia Salvador BA Brasil Faculdade de Medicina, Universidade Federal da Bahia, Salvador, BA, Brasil; 5 Escola Bahiana de Medicina e Saúde Pública Salvador BA Brasil Escola Bahiana de Medicina e Saúde Pública, Salvador, BA, Brasil

**Keywords:** Type 1 diabetes mellitus, cow’s milk, autoantigens, molecular mimicry

## Abstract

**Objective:**

To analyze in silico the evidence of molecular mimicry between human beta-cell autoantigens and cow’s milk proteins as a potential type 1 diabetes mellitus (T1DM) trigger.

**Materials and methods:**

The in silico analysis was performed using bioinformatics tools to compare the amino acid sequences of cow’s milk proteins (bovine serum albumin [BSA] and beta-lactoglobulin [BLG]) and human beta-cell autoantigens (glutamic acid decarboxylase-65 [GAD-65], insulin, and zinc transporter 8 [ZnT8]). The structural and functional characteristics of the proteins were analyzed to identify potential molecular mimicry mechanisms.

**Results:**

The results of the in silico analysis showed significant sequence similarity between BSA/BLG and GAD-65/human insulin/ZnT8, ranging from 19.64% to 27.27%. The cow’s milk proteins evaluated shared structural features with the beta-cell antigens selected for comparison, indicating a potential for molecular mimicry between these proteins.

**Conclusion:**

The findings of this study provide further evidence for a potential role of cow’s milk proteins in triggering T1DM. The in silico analysis suggests that molecular mimicry mechanisms between cow’s milk proteins and human beta-cell antigens may contribute to the autoimmune response leading to T1DM.

## INTRODUCTION

Molecular mimicry refers to the structural similarity of two or more molecules that may result in the human immune system mistaking a harmless substance for a pathogen. This is a common mechanism in the development of autoimmune diseases, where the immune system attacks healthy tissues and organs, leading to chronic inflammation and tissue damage (1).

Type 1 diabetes mellitus (T1DM) is an autoimmune disease caused by the destruction of pancreatic beta cells, resulting in insufficient production of insulin. The exact pathogenesis of T1DM remains unknown, but several factors have been implicated, including genetic predisposition, environmental triggers, and host immune factors (2,3). Recent studies have suggested a molecular mimicry mechanism as a potential environmental trigger for T1DM development. Cow’s milk proteins have been identified as potential trigger antigens in molecular mimicry involving glutamic acid decarboxylase-65 (GAD-65), human insulin, and zinc transporter 8 (ZnT8) in the development of T1DM (4). Cow’s milk is a complex mixture of proteins, lipids, and carbohydrates that serves as a source of nutrition for newborn calves. However, some of the proteins in cow’s milk have structural similarities to human proteins, which could trigger an autoimmune response in susceptible individuals. High levels of antibodies to cow’s milk proteins – such as bovine serum albumin (BSA) and beta-lactoglobulin (BLG) – have been associated with T1DM (5). Improved humoral immune response, characterized by increased IgG and IgA antibodies to protein fragments found in cow’s milk, have been described in individuals with a recent diagnosis of T1DM (6).

The existing bioinformatics software for conducting structural analysis of protein components found in cow’s milk is a crucial tool for assembling these proteins and comprehending their molecular mechanisms. Along the same lines, molecular modeling has aided in comprehending the concept of molecular mimicry, which occurs because of a cross-immune response to multiple antigens that resemble GAD-65, human insulin, and ZnT8 antigens. An essential prerequisite for effectively modeling similarity is the presence of a sufficiently similar protein sequence. Thus, the molecular mimicry between GAD-65/human insulin/ZnT8 and cow’s milk proteins is a well-documented phenomenon that may contribute to the pathogenesis of T1DM by inducing cross-reactive immune responses against beta-cell antigens. These observations highlight the importance of dietary interventions and antigen-specific therapies in the prevention and treatment of autoimmune diseases.

In this study, we analyzed *in silico* the evidence of molecular mimicry between GAD-65/human insulin/ZnT8 and cow’s milk proteins as a potential T1DM trigger.

## MATERIALS AND METHODS

To analyze the potential link between GAD-65/human insulin/ZnT8 and cow’s milk proteins as a trigger for T1DM, we used the Protein Data Bank (PDB) database to obtain the structural information of the proteins and the VectorBuilder website (https://en.vectorbuilder.com/) to evaluate the similarity between these proteins.

The first step was to obtain the crystal structure data of GAD-65, human insulin, ZnT8, and cow’s milk proteins (BSA and BLG) from the PDB database. Subsequently, the UniProt tool was used to visualize the sequence of these proteins.

We performed an immunoinformatic prediction of GAD-65, human insulin, ZnT8, and cow’s milk proteins epitopes using the NetMHCpan v4.1 software (https://services.healthtech.dtu.dk/service.php?NetMHCpan-4.1) (7), based on artificial neural networks, following the recommended methodology and filtering high-affinity epitopes based on an IC50 ≤ 50 nM and percentile rank ≤ 0.20 for the respective alleles analyzed. Subsequently, we performed a comparison of the epitopes to verify the hypothesis of cross-recognition. The epitope similarity analyses were performed online using VectorBuilder and complemented with Pairwise (https://www.rcsb.org/alignment), an extension of the PDB program, for conformational similarity analyses. NetMHCpan 4.1 is a server that uses artificial neural networks to filter high-affinity epitopes (8).

For the extraction of epitopes through NetMHCpan 4.1, we used the FASTA format obtained from the UniProt protein database website, selecting 11mer peptides and the human HLA-A*01:01 allele.

Pairwise structural alignment is a method of comparing two proteins by aligning their amino acid sequences and identifying similarities and differences in their structures. In the analysis of the beta-cell autoantigens (GAD-65, human insulin, and ZnT8) and cow’s milk proteins (BSA and BLG), we performed a pairwise alignment between the amino acid sequences of each protein to determine their degree of structural similarity. Once the sequences were obtained, pairwise structural alignment compared each amino acid in the two sequences to find matches and gaps in the sequence and then calculated the optimal alignment based on the highest number of matching amino acids.

The degree of similarity between the proteins was quantified based on the number and location of similar features in the structures. This method of analysis using pairwise structural alignment allows for a detailed comparison between two proteins and can provide insight into the similarities and differences in their structure and function.

For comparison of candidate epitopes, we used those that showed the strongest binding, programmed in NetMHCpan 4.1 between 0.5 and 2.0. The similarity between the sequences of GAD-65 epitopes, human insulin, and ZnT8 epitopes was evaluated using the UniProt BLAST tool.

To establish structural mimicry, we used UniProt’s Align tool, which is based on a simple approach of maximizing the TM score and the quantitative evaluation of the root-mean-square deviation (RMSD). The server is available for free at https://www.uniprot.org/align.

### Ethical aspects

According to Resolution CNS 510/2016, our study did not require approval from an ethics committee, since it aimed at deepening the theoretical understanding of situations that arise spontaneously and contingently in medical practice.

## RESULTS

For the prediction of epitopes for GAD-65, we selected four candidate epitopes, all of which exhibited a threshold indicating stronger binding peptides (weighted binding [WB] 0.693-1.313) (Table 1).

**Table 1:** Prediction of epitopes for GAD-65

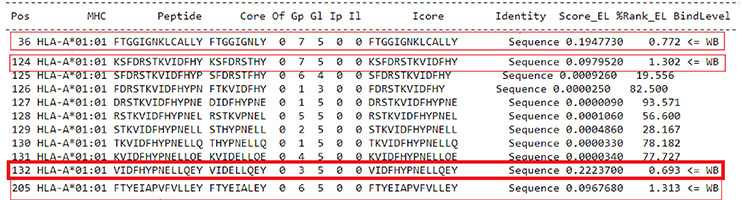

Source: NetMHCpan 4.1 (7).

For the prediction of epitopes for human insulin, two candidate epitopes were selected, both of which exhibited a threshold indicative of stronger binding peptides (WB 1.153-1.892) (Table 2).

**Table 2:** Prediction of epitopes for human insulin

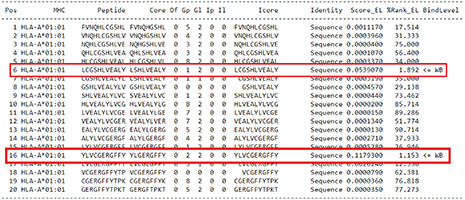

Source: NetMHCpan 4.1 (7).

For the prediction of epitopes for ZnT8, four candidate epitopes were selected, two of which exhibited a threshold of stronger binding peptides (WB 0.346-1.984) (Table 3).

**Table 3:** Prediction of epitopes for ZnT8

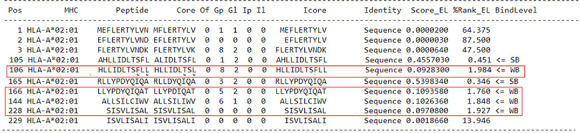

Source: NetMHCpan 4.1 (7).

For the prediction of epitopes for BSA, four candidate epitopes were selected, all of which exhibited a threshold indicative of stronger binding peptides (WB 0.587-1.987) (Table 4).

**Table 4:** Prediction of epitopes for BSA

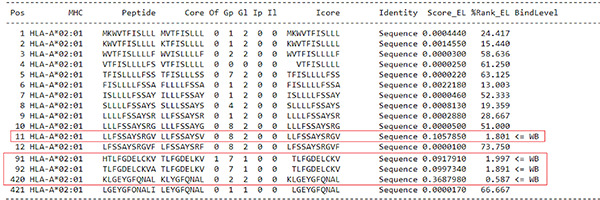

Source: NetMHCpan 4.1 (7).

For the prediction of epitopes for BLG, four candidate epitopes were selected, all of which exhibited a threshold indicative of stronger binding peptides (WB 0.587-1.987) (Table 5).

**Table 5:** Prediction of epitopes for BLG

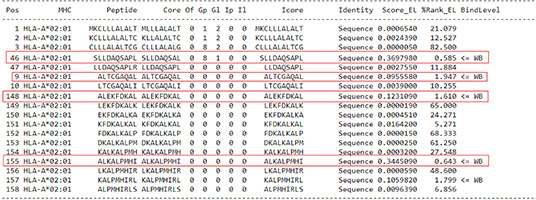

Source: NetMHCpan 4.1 (7).

### Analyses of epitope similarities between the human beta-cell antigens and cow’s milk proteins

**Figure 1:**
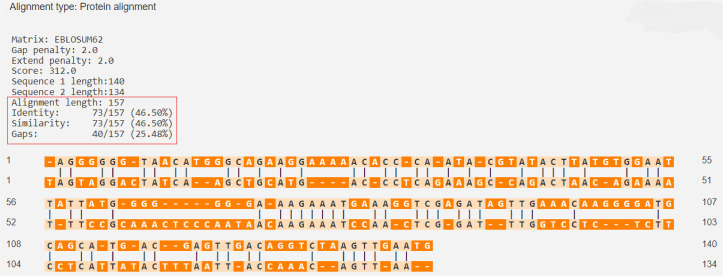
GAD-65 (Q05329 · DCE2_HUMAN) versus BSA (P02769 · ALBU_BOVIN).

Source: https://en.vectorbuilder.com/tool/sequence-alignment.html.

**Figure 2:**
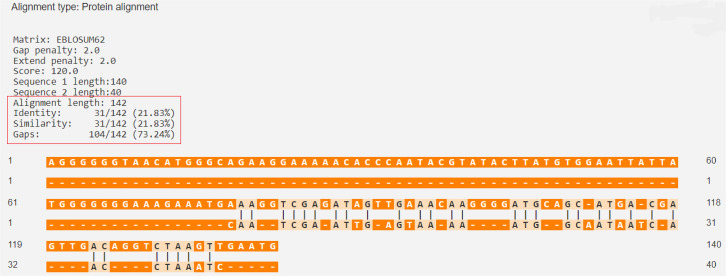
GAD-65 (Q05329 · DCE2_HUMAN) versus BLG (P02754 · LACB_BOVIN).

Source: https://en.vectorbuilder.com/tool/sequence-alignment.html.

The percentage of similarity between the strongest binding GAD-65 epitope and the BSA epitopes was 46.50%, while the percentage of similarity between the strongest binding GAD-65 epitope and the BLG epitopes was 21.83% (Figures 1 and 2).

**Figure 3:**
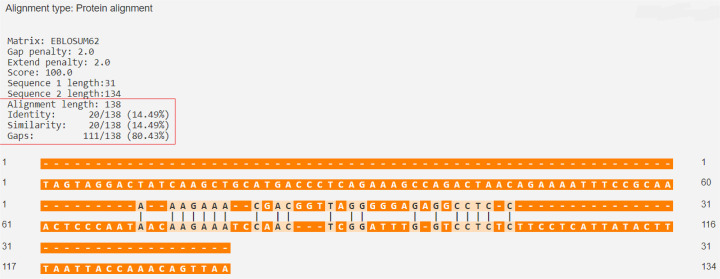
Human insulin (P01308 · INS_HUMAN) versus BLG (P02754 · LACB_BOVIN).

Source: https://en.vectorbuilder.com/tool/sequence-alignment.html.

**Figure 4:**
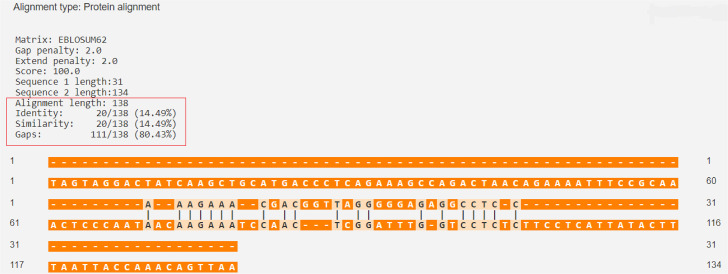
Human insulin (P01308 · INS_HUMAN) versus BSA (P02769 · ALBU_BOVIN).

Source: https://en.vectorbuilder.com/tool/sequence-alignment.html.

The percentage of similarity between the strongest binding human insulin epitope and the BSA epitopes was 14.49%, while the percentage of similarity between the strongest binding human insulin epitope and the BLG epitopes was 14.49% (Figures 3 and 4).

**Figure 5:**
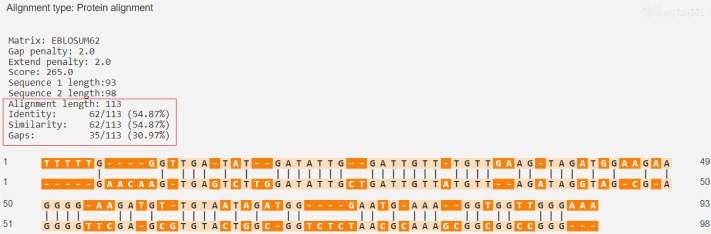
ZnT8 (Q8IWU4 · ZNT8_HUMAN) versus BLG (P02754 · LACB_BOVIN).

Source: https://en.vectorbuilder.com/tool/sequence-alignment.html.

**Figure 6:**
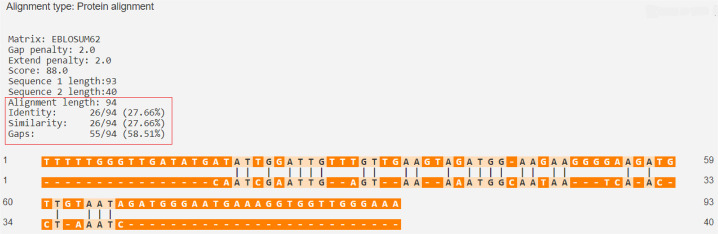
ZnT8 (Q8IWU4 · ZNT8_HUMAN) versus bovine serum albumin (P02769 · ALBU_BOVIN)

Source: https://en.vectorbuilder.com/tool/sequence-alignment.html.

The percentage of similarity between the strongest binding ZnT8 epitope and the BSA epitopes was 54.87%, while the percentage of similarity between the strongest binding human insulin epitope and the BLG epitopes was 27.66% (Figures 5 and 6).

The conformational similarity analysis between cow’s milk proteins and GAD-65/human insulin/ZnT8 proteins involved comparing their structures to identify homology and structural similarities. By evaluating the degree of structural overlap and comparing key interaction residues, we were able to gain insights into potential functional and immunological implications. This allowed us to explore potential cross-reactivity and identify structural similarities that may be involved in autoimmune responses or other biological interactions related to the onset of T1DM, as demonstrated below. Our results from the conformational similarity analysis between cow’s milk proteins and GAD-65/human insulin/ZnT8 proteins showed a structural comparison.

### Analysis of conformational similarities between the human beta-cell antigens and cow’s milk proteins

**Figure 7:**
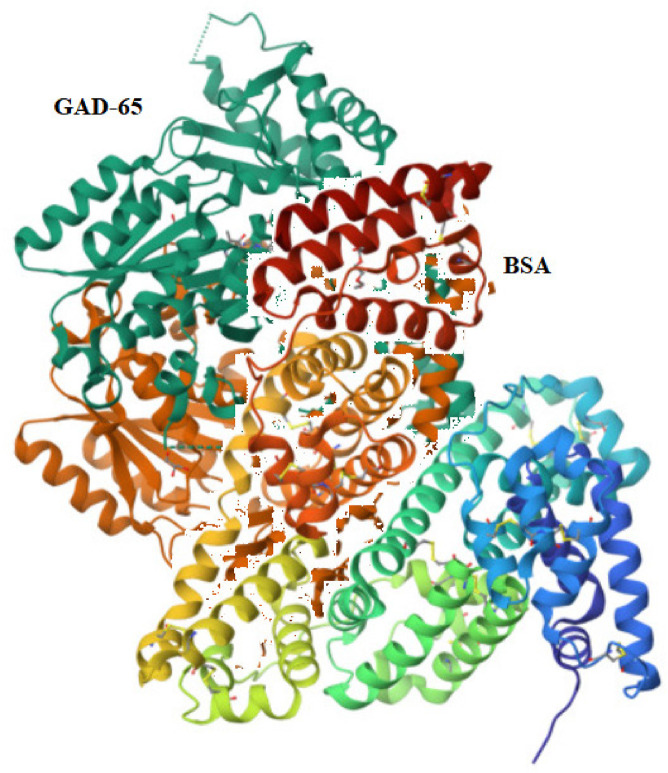
Structure alignment analysis between GAD-65 and BSA.

Source: https://www.rcsb.org/alignment.

The pairwise structure alignment analysis showed a sequence similarity between GAD-65 and BSA, with a 46.50% identity (Figure 7).

**Figure 8:**
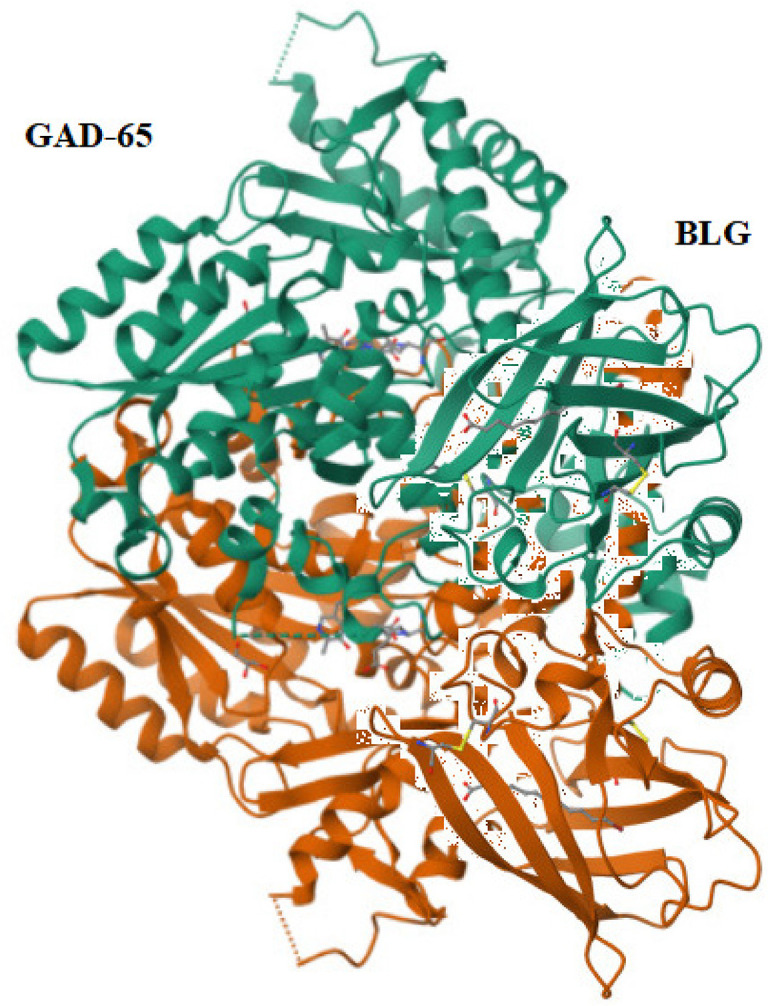
Structure alignment analysis between GAD-65 and BLG.

Source: https://www.rcsb.org/alignment.

The pairwise structure alignment analysis showed a sequence similarity between GAD-65 and BLG, with a 40.00% identity (Figure 8).

**Figure 9:**
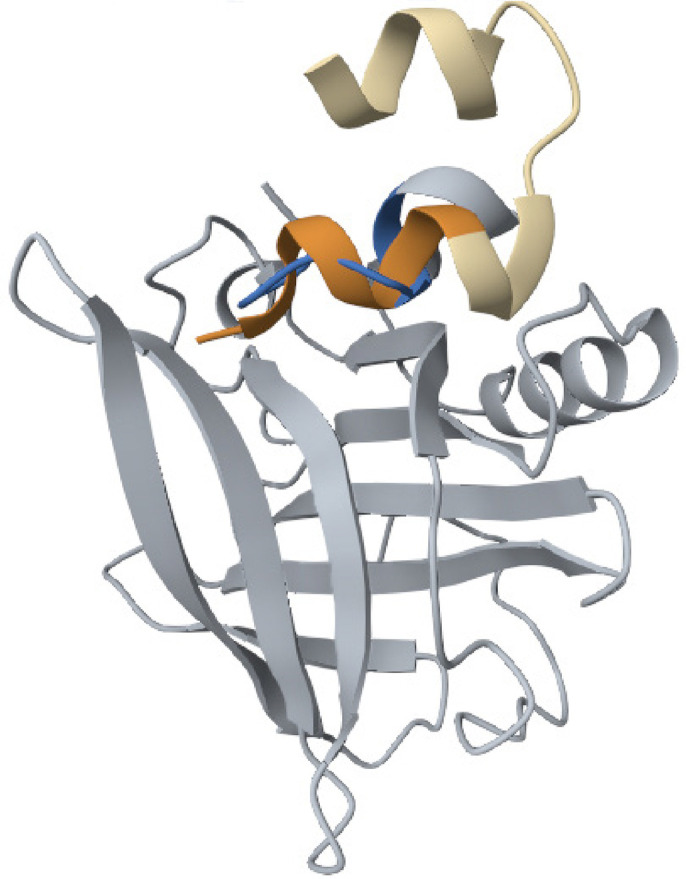
Structure alignment analysis between human insulin and BLG.

Source: https://www.rcsb.org/alignment.

The pairwise structure alignment analysis showed a significant sequence similarity between the human insulin and BLG, which shared 57.00% of sequence identity (Figure 9).

**Figure 10:**
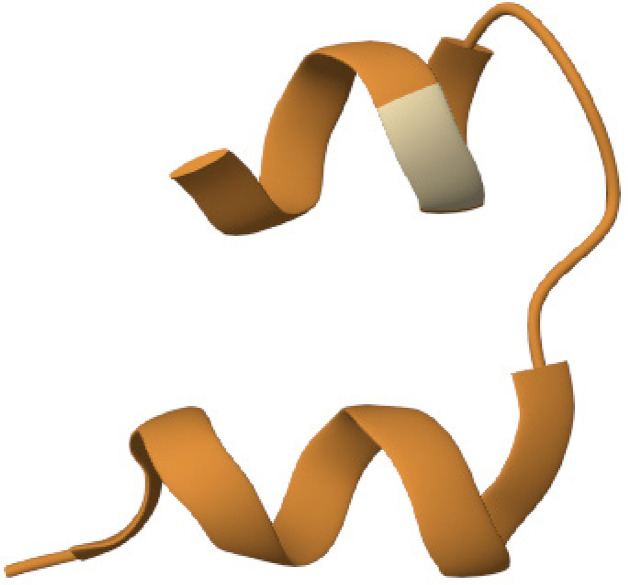
Structure alignment analysis between human insulin and BSA.

Source: https://www.rcsb.org/alignment.

The pairwise structure alignment analysis showed structural similarity between human insulin and BSA, which shared 33.00% of sequence identity (Figure 10).

Human insulin and BSA shared structural similarities in their tertiary structures, as both proteins have alpha helices and beta sheets. Additionally, both proteins have a similar fold in their binding domains. Overall, they have a RMSD value of 2.00 Å, indicating good similarity.

**Figure 11:**
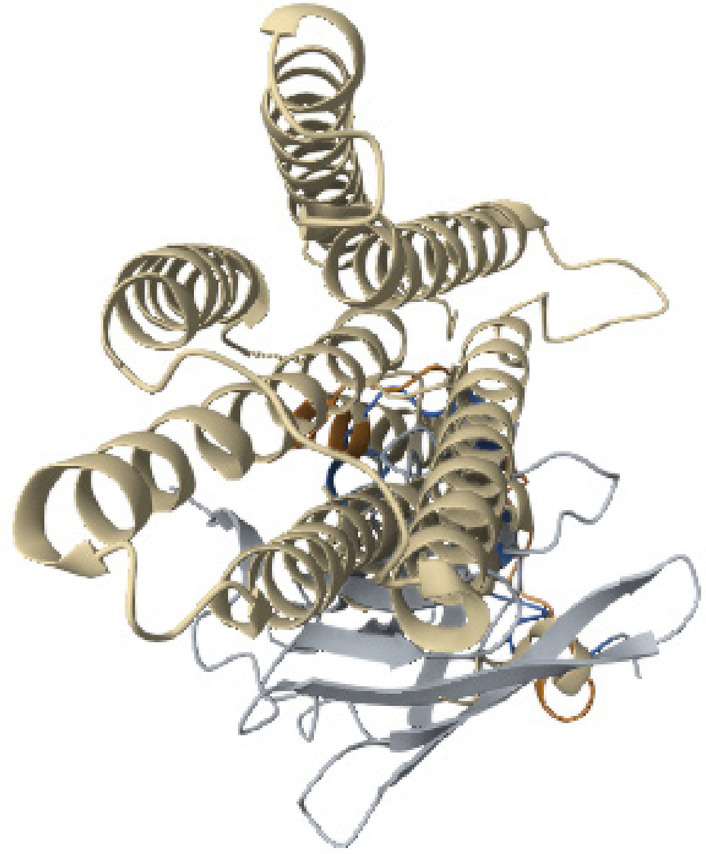
Structure alignment analysis between ZnT8 and BLG.

Source: https://www.rcsb.org/alignment.

**Figure 12:**
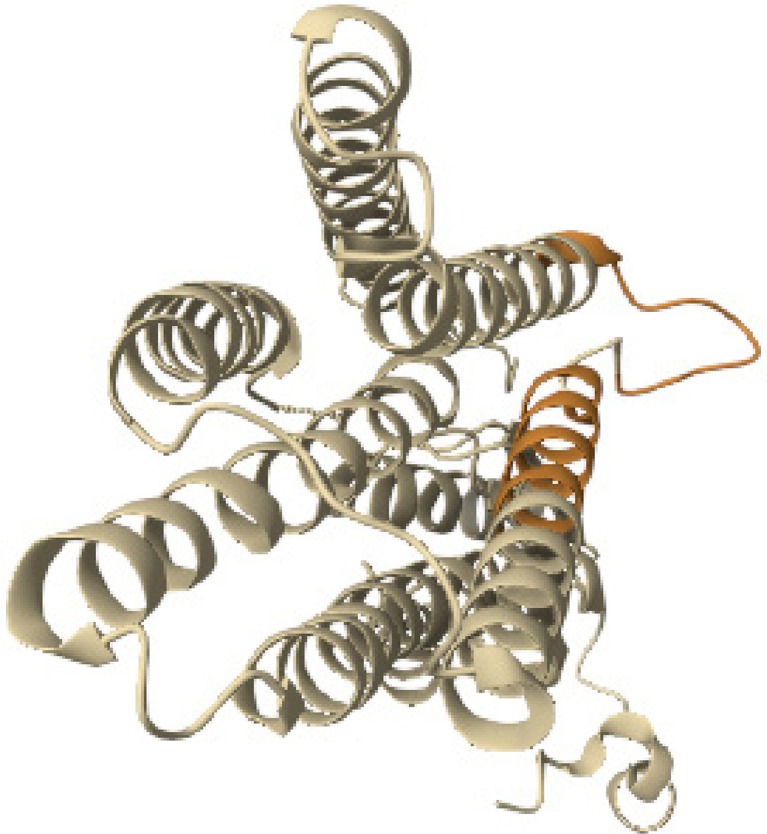
Structure alignment analysis between ZnT8 and BSA.

Source: https://www.rcsb.org/alignment.

The pairwise structure alignment analysis showed a significant sequence similarity between the ZnT8 and BLG, which shared 41.00% sequence identity (Figure 11). The structural similarity between human insulin and BSA showed 18.29% sequence identity (Figure 12).

Despite potential sequence differences, conformational similarity and sequence identity provided valuable information. Although amino acid sequences may differ between pairs of proteins, and a simple analysis of the amino acid sequences may result in false-positive results, we did not exclude the analysis of similarity between cow’s milk proteins and GAD-65/human insulin/ZnT8 proteins.

## DISCUSSION

The molecular mimicry mechanism between cow’s milk proteins and human insulin and GAD-65 provides a potential environmental trigger for T1DM development in genetically predisposed individuals. The present study investigated the possibility of the cow’s milk proteins BSA and BLG sharing homology with GAD-65, human insulin, and ZnT8 and causing T1DM.

In 1987, Damian proposed that the resemblance of an antigen protein to that of the host was a mechanism to evade recognition and elimination by the host (9). This phenomenon, known as molecular mimicry, may contribute to autoimmune diseases by triggering an adverse autoimmune response through cross-reactivity between protein epitopes and antigens present in the body (10).

The investigation of molecular mimicry using bioinformatics in autoimmune diseases plays a crucial role in the understanding and potential treatment of these complex conditions. By utilizing computational tools and techniques, researchers can analyze and identify similarities between self-antigens and microbial antigens, which may trigger autoimmune responses. Bioinformatics approaches offer a comprehensive and systematic examination of the immune system, enabling the identification of potential molecular mimics and their impact on autoimmunity. Recent studies have highlighted the significance of bioinformatics-based approaches in unraveling the molecular mechanisms underlying autoimmune diseases and discovering novel therapeutic targets (11,12). These findings emphasize the need for further research and collaboration in this field to improve diagnostic accuracy and develop personalized treatment strategies for individuals affected by autoimmune disorders.

One of the environmental factors that have been implicated in the development of T1DM is cow’s milk. Cow’s milk contains several proteins that may trigger an autoimmune response in susceptible individuals. Two of these proteins are BLG and BSA (13).

Several studies have investigated the role of molecular mimicry between cow’s milk proteins and GAD-65/human insulin/ZnT8 in the development of T1DM. Some studies have reported that cow’s milk consumption during infancy is associated with an increased risk of developing T1DM later in life, while others have failed to find a significant association. One of the limitations of these studies is the difficulty in establishing an accurate measure of cow’s milk consumption. Cow’s milk is often used as an ingredient in many processed foods, hindering an accurate assessment of the amount of cow’s milk consumed by an individual.

One of the earliest studies on molecular mimicry in T1DM was conducted by Elliott and cols. (14). The authors used monoclonal antibodies to examine the cross-reactivity between GAD-65 and several proteins, including BSA and BLG, the two most abundant proteins in cow’s milk. The study found that some of the monoclonal antibodies created against BSA and BLG recognized GAD-65, indicating a structural similarity between the two proteins. However, the authors did not investigate the clinical implications of this observation.

Another study provided evidence for the role of molecular mimicry in the development of T1DM. The authors demonstrated that feeding cow’s milk formula to infants who carried the HLA-DQB1*0201 allele, a genetic risk factor for T1DM, increased the risk of developing islet autoantibodies. They also showed that the autoantibodies recognized an epitope in BSA that shared structural similarity with GAD-65. The authors concluded that cow’s milk proteins could induce the production of autoantibodies against GAD-65 through molecular mimicry, which could contribute to the development of T1DM (15).

Notably, BSA has been shown to share structural similarities with GAD-65 (16). The molecular mimicry between cow’s milk proteins and GAD-65 has been suggested as another potential trigger of T1DM. As an enzyme in pancreatic beta cells, GAD-65 catalyzes the conversion of glutamate to gamma-aminobutyric acid. Additionally, GAD-65 is a major autoantigen in T1DM, and autoantibodies to GAD-65 are detected in 60–70% of individuals with newly diagnosed T1DM, suggesting that the destruction of beta cells may be mediated by the immune response against GAD-65 (17).

Despite their different functions and structures, BSA and GAD-65 share a certain degree of amino acid similarity. Several studies have reported sequence alignments and comparisons between BSA and GAD-65 using bioinformatics tools. A study using multiple sequence alignment to compare the amino acid sequences of BSA and GAD-65 found that the two proteins presented 29% sequence identity and 48% sequence similarity, with most of the similar residues located in the flexible loops and turns of the proteins (18). Our study, using pairwise structure alignment analysis, compared the amino acid sequences of BSA and GAD-65. The evaluation between these two proteins showed a lower percentage of sequence similarities than other studies in the literature, with most of the similar residues located in the flexible loops and bends of the proteins. In addition, we used homology modeling to generate a three-dimensional structure of BSA and compared it to the crystal structure of GAD-65. Our results showed that BSA shares an overall fold similar to that of GAD-65, which has not been described in previous studies.

We found no studies in the literature evaluating the similarity between GAD-65 and BLG. In our study, we compared the amino acid sequences of BLG and GAD-65, which showed 21.83% sequence identity and 40.00% sequence similarity between these two proteins.

Insulin and BLG, despite their distinct functions, share a resemblance in their amino acid sequences. Several studies have suggested that the amino acid sequence of BLG contains regions that resemble the amino acid sequence of human insulin, thereby leading to molecular mimicry. Molecular mimicry occurs when a foreign antigen (in this case, BLG) shares structural similarities with a self-antigen (in this case, human insulin), leading to cross-reactivity of T cells and autoimmunity (19).

Several *in silico* studies have evaluated the molecular mimicry between insulin and cow’s milk proteins in triggering T1DM. These studies aimed to investigate the structural and immunological similarities between the two proteins and their potential role in the development of autoimmune reactions leading to diabetes. Previous studies have shown that antibody cross-reactivity between human insulin and BLG is possible, indicating that molecular mimicry may be a contributing factor (20). However, we found no *in silico* studies in the literature comparing the similarity between BLG and human insulin. Our study using pairwise structure alignment analysis compared the amino acid sequences of BLG and human insulin and found that these two proteins share a 57.00% sequence identity and 14.49% sequence similarity and structurally show a required minimum distribution indicative of good similarity.

Reviewing the medical literature, we found no studies in which the similarity between human insulin and BSA was assessed. In our study, we compared the amino acid sequences of human insulin and BSA and found a sequence identity of 33.0% and a sequence similarity of 14.49% between these two proteins.

An analysis focused on discovering beta–cell-specific proteins associated with the regulatory pathway of secretion recently identified ZnT8 as a novel autoantigen (21). Found in the membrane of insulin-containing secretory granules, ZnT8 is responsible for transporting zinc ions from the cytosol into the vesicles (22). Additionally, ZnT8 has emerged as a potential target for humoral autoimmunity, making it highly relevant for the early detection of T1DM. The presence of ZnT8-specific CD8+ T cells can be observed in the majority of T1DM patients, and these cells play a crucial role in the development of T1DM. As a target for immunotherapy, ZnT8 offers the potential to ameliorate beta-cell dysfunction in T1DM, presenting new avenues for the treatment of this condition (23).

It has been reported that anti-ZnT8 antibodies that target homologous membrane extension sequences exhibit cross-reactivity and have the ability to elicit robust immune responses, thus raising the possibility of a molecular mimicry mechanism that triggers T1DM (24). The literature suggests that infection with the *Mycobacterium avium* subspecies paratuberculosis is linked to T1DM by producing anti-ZnT8 antibodies via molecular homology (25). However, there are no studies available related to molecular mimicry between cow’s milk proteins and ZnT8. Our study compared the amino acid sequences of BSA and ZnT8. The evaluation between these two proteins showed 54.87% sequence identity and 18.29% sequence similarity. When comparing the amino acid sequences of ZnT8 and BSA, we found a sequence identity of 41.00% and a sequence similarity of 27.66% between these two proteins.

Molecular modeling has enabled the comprehension of molecular mimicry caused by the cross-immune reaction to cow’s milk protein-like antigens (BSA/BLG) with GAD-65/human insulin/ZnT8. The majority of molecular mimicry probably includes T-cell mediation, as T cells typically detect linear peptides that vary from 8 to 20 amino acids long (26).

Post-translational modifications refer to chemical alterations that occur in a protein after the translation of its genetic code. These modifications can involve the addition, removal, or alteration of functional groups in specific amino acid residues of the protein (27). Post-translational modifications play a crucial role in regulating the structure, function, and subcellular localization of proteins. They can influence enzymatic activity, protein stability, interaction with other proteins, and even cellular signaling (28). The analysis of post-translational modifications was not utilized in our study due to its focus on the primary sequence of the protein and its three-dimensional structure. In these cases, obtaining information about the specific composition of amino acids and their spatial organization is more important than investigating the chemical alterations that occur after genetic code translation.

A study *in silico*, similar to ours, demonstrated that BSA and BLG share sequence and structural homology with GAD-65 (16). However, our study is more comprehensive in terms of the larger number of proteins evaluated compared with the cited study. Investigations in molecular mimicry using bioinformatics are critically important in the understanding and management of autoimmune diseases. Bioinformatics approaches enable the identification and analysis of these mimetic epitopes, providing insights into the mechanisms underlying autoimmunity and aiding in the development of targeted therapies. Studies have shown the utility of bioinformatics in unraveling molecular mimicry in diseases (29). Thus, by elucidating the molecular basis of autoimmune reactions, these studies pave the way for precision medicine approaches and personalized therapeutic interventions.

Although similarities have been detected between BSA, BLG, GAD-65, human insulin, and ZnT8, a randomized clinical trial including 2,159 children from 15 countries explored the impact of excluding cow’s milk proteins from infant formula on the development of T1DM in high-risk infants. The study found that removing cow’s milk proteins from infant formulas did not prevent the development of T1DM in these high-risk children (30). This randomized international study included children with an HLA genotype associated with increased risk of T1DM and a first-degree relative affected by the disease were included. The majority of participants came from Canada, Finland, and the United States. The larger sample size of this study provided greater statistical power compared with the pilot study (15), allowing for a more definitive conclusion on whether transitioning to an extensively hydrolyzed formula protects against diabetes. The study was designed to have two endpoints, specifically, the presence of two autoantibodies at age 6 and clinical T1DM at age 10. However, it is important to note that the results cannot be directly applied to the general population, as the participants were selected based on a positive family history and a specific HLA genotype associated with the risk of T1DM. Moreover, these findings may not be applicable to children with different HLA genotypes. However, it is worth noting the contrasting results obtained when compared with those of other studies (31-35).

In view of the above, we must consider the several limitations of *in silico* studies that focus on identifying how molecular mimicry between human pancreatic beta-cell antigens and cow’s milk proteins may trigger T1DM. First, the molecular mimicry mechanism is not the only environmental trigger for T1DM development. Other factors, such as viral infections, gut microbiota, and dietary factors, may also contribute to T1DM pathogenesis (36). Second, the evidence for the molecular mimicry between cow’s milk proteins and human insulin, GAD-65, and ZnT8 is mainly based on *in vitro* studies, and the clinical significance of these findings is still unclear.

In conclusion, the *in silico* analysis in the present study suggests that molecular mimicry mechanisms between proteins from cow’s milk and human pancreatic beta-cell antigens may contribute to the autoimmune response that may trigger T1DM.
